# Early Echocardiographic and Serum Biomarkers Predict Thrombotic Microangiopathy, Endotheliopathy, and Survival After Pediatric Hematopoietic Stem Cell Transplant

**DOI:** 10.1016/j.jtct.2025.11.024

**Published:** 2025-11-15

**Authors:** Michael A. Smith, Zachary Hutchinson, Elizabeth Colglazier, Claire Parker, Christine S. Higham, Jeffrey R. Fineman, Matt S. Zinter, Christopher C. Dvorak, Hythem M. Nawaytou

**Affiliations:** 1 Division of Pediatric Critical Care, Department of Pediatrics, University of California, San Francisco, California; 2 Pediatric Pulmonary Hypertension Program, Department of Pediatrics, University of California, San Francisco, California; 3 Division of Pediatric Cardiology, Department of Pediatrics, University of California, San Francisco, California; 4 Division of Allergy, Immunology, and Bone Marrow Transplantation, Department of Pediatrics, University of California, San Francisco, California

**Keywords:** Pediatric hematopoietic, stem cell transplant, Transplant-associated, thrombotic, microangiopathy, endotheliopathy, Cardio-oncology

## Abstract

Many life-threatening complications of hematopoietic stem cell transplantation (HSCT) develop secondary to endothelial injury and dysfunction, including transplant-associated thrombotic microangiopathy (TA-TMA), sinusoidal obstruction syndrome, idiopathic pneumonia syndrome, and engraftment syndrome. These endotheliopathies are often accompanied by cardiovascular compromise. Echocardiographic abnormalities including pericardial effusions and elevated right ventricular pressure have previously been identified as early indicators of TA-TMA. Additional echo-derived parameters and serologic metrics of ventricular function are yet to be evaluated as predictors of post-HSCT endotheliopathies and mortality among children. We sought to assess the utility of early post-HSCT echo and B-type natriuretic peptide (BNP) screening in predicting the development of TA-TMA, additional endotheliopathies, and death. A single-center, prospective cohort study was performed after the implementation of a uniform screening protocol at our pediatric hospital. Patients who received a HSCT from October 2021 to August 2023 were screened with echocardiography and serum BNP levels pre-HSCT, d +7 and d +30 from transplant. Changes from baseline in echocardiographic metrics of right and left ventricular function, pericardial effusions, and BNP levels were evaluated as predictors of post-HSCT TA-TMA, additional endotheliopathies, and death. Fifty-two patients underwent a first HSCT during the study period. The 1-yr cumulative incidence of TA-TMA was 13.8% ± 9.6%, of any endotheliopathy was 39.2% ± 13.7% and of death was 13.5% ± 9.4%. Several echocardiographic predictors were found to be associated with the later development of TA-TMA, including pericardial effusions (HR 7.59, 95% CI: 1.80 to 32.00, *P* = .006) and measures of increased right and left ventricular function, such as a 10% increase in longitudinal tricuspid annular systolic velocity (tricuspid s’, HR 1.61, 95% CI: 1.08 to 2.38, *P* = .018), and a 10% increase in mitral s’ (HR: 1.70, 95% CI: 1.05 to 2.75, *P* = .031) evaluated at d +30. Similarly, several echocardiographic metrics were associated with the later development of any endotheliopathy, including measures of increased ventricular function such as a 10% increase in tricuspid annular plane systolic excursion (HR 1.43, 95% CI: 1.08 to 1.90, *P* = .013) and a 10% increase in mitral s’ (HR: 1.28, 95% CI: 1.05 to 1.57, *P* = .016) at d +30, as well as a BNP increase of 50pg/ml from baseline at d +7 (HR: 2.86, 95% CI: 1.15 to 7.08, *P* = .024). Mortality was significantly increased for patients with at least a 10% increase in left ventricular ejection fraction at d +7 (*P* = .035) and pericardial effusions (*P* = .002). Among pediatric HSCT recipients, acute cardiovascular injury associated with HSCT-related endotheliopathy develops at a subclinical level in the early post-transplant period. Subtle increases in right and left ventricular function, increases in BNP level, and pericardial effusions likely reflect an early response to endothelial injury and are associated with the later development of TA-TMA, other endotheliopathies, and death. Screening protocols and prophylactic and therapeutic interventions for pediatric HSCT recipients should consider these novel cardiovascular biomarkers.

## INTRODUCTION

Transplant-associated thrombotic microangiopathy (TA-TMA) is a severe complication of hematopoietic stem cell transplantation (HSCT) driven by endothelial injury, which leads to microangiopathic hemolytic anemia, thrombotic ischemia, and multiorgan dysfunction [1–5]. Endothelial dysfunction is a common pathophysiologic mechanism underlying a number of additional post-HSCT complications, including hepatic sinusoidal obstruction syndrome (SOS), idiopathic pneumonia syndrome (IPS), and engraftment syndrome (ES) [6,7]. Cardiovascular compromise is often an early and prominent feature of these endotheliopathies [8].

Prior studies have evaluated the associations between several echocardiographic abnormalities and these complications. TA-TMA, specifically, has been associated with pericardial effusions, elevated right ventricular (RV) pressure, and left ventricular (LV) dysfunction [9–16]. Pericardial effusions develop relatively frequently after HSCT. Rotz et al identified pericardial effusions in 57/209 (27%) pediatric patients within the first 100 d after HSCT, with a significantly higher incidence noted among those with TA-TMA [13]. Elevated RV pressure secondary to pulmonary hypertension (PH) is a potentially lethal complication of HSCT, with varying reports of its incidence but multiple reports of an association with TA-TMA [10,11,14,15]. LV dysfunction may develop secondary to cardiotoxic therapies received prior to HSCT as well as in conjunction with TA-TMA and other endotheliopathies. In one cohort, LV dysfunction was noted in 22/70 (31%) of pediatric patients admitted to the pediatric intensive care unit after HSCT, many of whom also had pericardial effusions and TA-TMA [16].

Risk stratification and early identification of TA-TMA and other endotheliopathies are key to mitigating their negative impact on outcomes. We hypothesized that cardiovascular complications diagnosed by echocardiography and B-natriuretic peptide (BNP) levels may aid in the prediction and early detection of post-HSCT endothelial dysfunction. As such, we implemented a uniform screening protocol for all pediatric HSCT recipients that included pre- and early post-HSCT echocardiograms and serum BNP levels. Here we report the results of this prospective study assessing the utility of this screening protocol in predicting post-HSCT endotheliopathies and death.

## METHODS

### Screening Protocol

We developed a uniform screening guideline for all recipients of HSCT at our pediatric institution. Screening consisted of an echocardiogram and a BNP level performed within 30–90 d before HSCT (pre-HSCT), an echocardiogram and BNP level at 7 d after HSCT (post-HSCT +7), and an echocardiogram at d 30 after HSCT (post-HSCT +30). Screening studies were obtained regardless of clinical status at the predetermined times. Additional echocardiograms and/or BNP levels were obtained when clinically indicated for patients admitted to the intensive care unit for cardiopulmonary indications and/or sepsis as well as those with new cardiopulmonary symptoms, including the need for supplemental oxygen for >24 h not related to mucositis. These results, as well as additional clinically indicated echocardiograms and BNP levels obtained at the discretion of the clinical team, were not included in subsequent analyses of the screening protocol. However, they were used to define the incidence and course of cardiovascular complications. Any abnormal results were referred to the pediatric cardiology and/or pediatric pulmonary hypertension services and additional diagnostics and management were determined on an individualized basis.

Echocardiograms were analyzed for evidence of pericardial effusion, pulmonary hypertension, and ventricular dysfunction. Pericardial effusion was graded subjectively as trivial, small, moderate, or large by 1 operator (YZ) who was blinded to patient outcomes. Metrics of pulmonary hypertension and ventricular function are summarized in Supplementary Figure 1. Pulmonary hypertension was defined as the presence of a tricuspid valve regurgitation jet peak velocity >2.8 m/sec or systolic septal flattening defined as an eccentricity index >1.2. Pulmonary artery acceleration time (PAAT) and its heart rate correction, PAAT divided by right ventricular ejection time (RVET), were evaluated as markers of pulmonary vascular resistance, with a value <0.3 considered abnormal [17]. The right ventricular systolic function was assessed using the tricuspid annular plane systolic excursion (TAPSE) measured in millimeters, its normalized version for age and sex (z score) [18], and lateral tricuspid annulus peak systolic velocity (tricuspid s’) by tissue Doppler. Right ventricular fractional area change and right ventricular strain were not analyzed due to inadequate imaging on baseline echocardiograms to perform these measurements. The left ventricular systolic function was assessed using the biplane Simpson method for calculating ejection fraction (LV EF), lateral mitral annulus peak systolic velocity (mitral S’) by tissue Doppler, and using longitudinal strain measured by speckle tracking in the apical 4-chamber view. The diastolic function of the right and left ventricles was evaluated using tricuspid and mitral annular velocities by tissue Doppler and the tricuspid and mitral inflow velocities by spectral Doppler, respectively. The ratio of early inflow velocity (E) to late inflow velocity (A) and the ratio of the early inflow velocity (E) to early annular velocity (e’) were used as surrogates for diastolic function.

### Outcomes

The primary outcome assessed was the development of TA-TMA within 1 yr of HSCT. We additionally assessed a composite outcome of any post-HSCT endotheliopathy, consisting of TA-TMA, SOS, IPS, and/or ES, as well as all-cause mortality. Diagnoses were determined from review of documentation and thus were made at the discretion of the on-service clinical teams. At our institution, TA-TMA is diagnosed using the harmonized Jodele criteria [19] and SOS is determined using the Cairo criteria [20]. IPS is considered a diagnosis of exclusion for those who develop respiratory distress and interstitial lung disease within 120 d of transplant and have negative microbiologic work-up. Engraftment syndrome is considered when patients develop fever, rash, and/or pulmonary infiltrates unrelated to infection within 5 d of engraftment. Additional clinical data, including infectious complications and need for invasive mechanical ventilation, renal replacement therapy, or pericardiocentesis, were also collected.

### Predictors of TA-TMA and Outcome

There are no universally accepted metrics to define cardiac complications in patients undergoing HSCT. Hence, we defined our own predictors using echocardiographic metrics and BNP levels. Complications in HSCT can present early and evolve quickly. Thus, our predictors were designed to be more sensitive than specific to allow for early intervention. The predictors included the presence of pericardial effusion or the presence of PH by echocardiography, as defined above. The American Society of Echocardiography guidelines on the evaluation of ventricular function after chemotherapy suggest that a less than 8% decrease in global longitudinal strain values from baseline is not meaningful, and a more than 15% change is clearly abnormal [21]. Taking this into consideration and our local echocardiography lab interobserver variability results, we defined a minimum of 10% change from baseline ventricular function metrics as predictors of myocardial involvement. Pericardial effusions on either screening echo were assessed for associations as long as they were first noted prior to onset of the outcomes of interest. For BNP levels, we defined a 50 pg/mL change from baseline, twice the expected weekly biologic variability in BNP levels in our cohort [22], as the predictor cutoff.

### Statistical Analysis

Standard descriptive statistics were used to describe patient demographic and clinical characteristics, including the primary outcomes and predictors of interest. Hazard ratios to describe the risk of TA-TMA and any endotheliopathy were determined from univariate competing risk regression models, treating death as a competing event and only incorporating predictor variables measured prior to the onset of the outcome being assessed. Univariate Cox regression models were used to derive hazard ratios (HR) and 95% confidence intervals (CI) describing the risk of mortality associated with each predictor. Logistic regression was then used to build models predicting the outcome of any endotheliopathy, using forward selection to first assess right ventricular (TAPSE + tricuspid S’) and left ventricular (LVEF + mitral S’) function followed by multivariable models combining biventricular function metrics, pericardial effusion, and change in BNP level. The diagnosis of any endotheliopathy was ensured to have occurred after the measurement of the predictors included in the models. Predictive performance was assessed via the area under receiver operating characteristic curves (AUC) and model calibration was assessed using the integrated Brier score (IBS) determined at median follow up. Lastly, Kaplan-Meier curves were used to demonstrate differences in survival after screening echocardiogram stratified by echocardiographic metrics, with comparisons between groups made via the Log Rank test.

## RESULTS

### Patient Characteristics and Outcomes

Between December 1, 2021 and August 1, 2023, 63 patients underwent HSCT out of which 52 patients completed the screening protocol. Eleven patients were excluded due to missing either the baseline or both post-HSCT echocardiograms. Patient demographics and transplant characteristics are described in Table 1.

**Figure 1.**
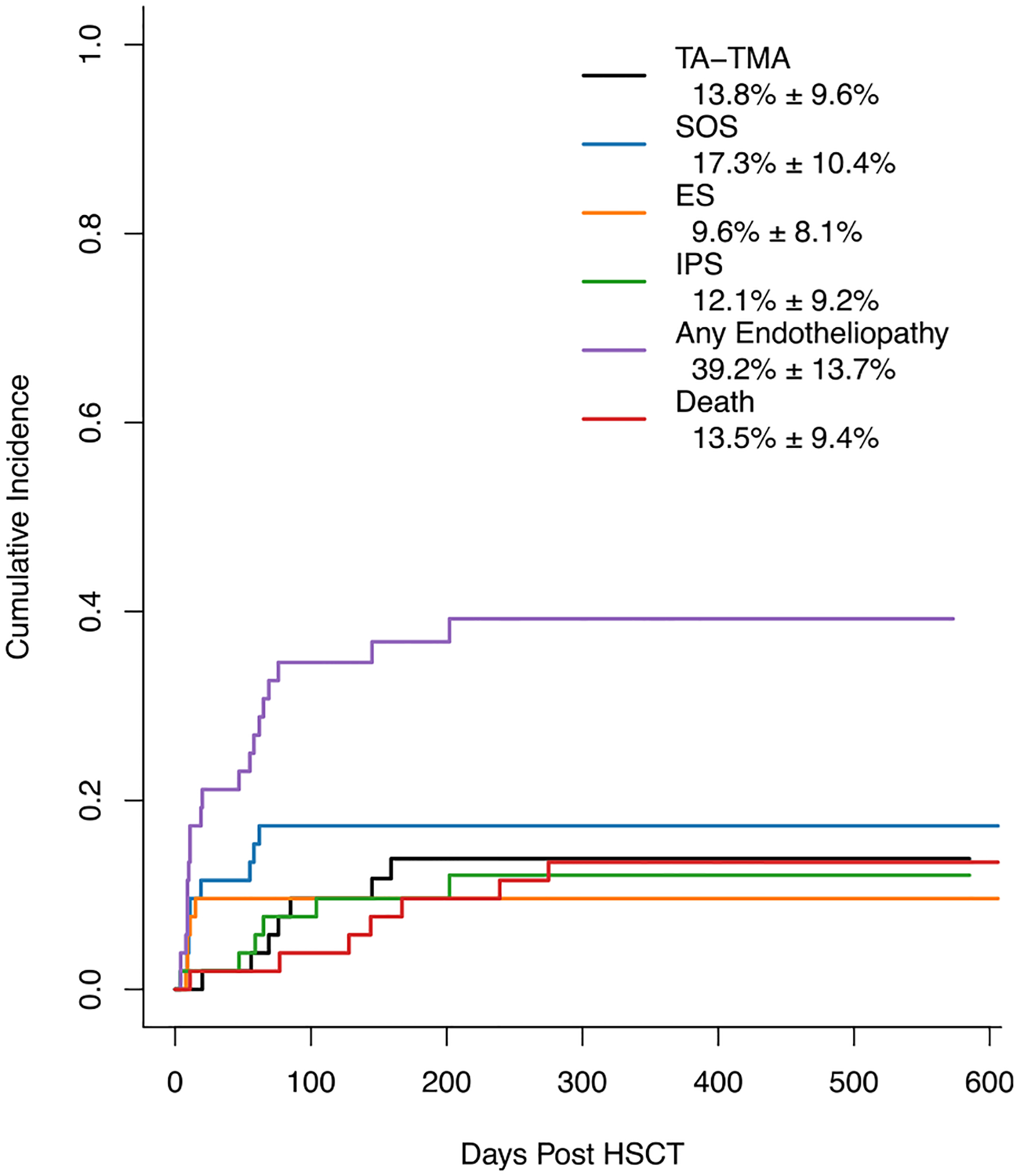
Cumulative incidence plots of primary and secondary outcomes Cumulative incidence plots of transplant-associated thrombotic microangiopathy (TA-TMA), sinusoidal obstruction syndrome (SOS), engraftment syndrome (ES), idiopathic pneumonia syndrome (IPS), any endotheliopathy, and death. Cumulative incidence estimates at 1-year post-hematopoietic stem cell transplant (HSCT) accounting for death as a competing risk are noted.

Patients were followed post-HSCT for a median of 11.6 months (interquartile range 7.8 to 16.8 months). Most patients were transplanted for malignant diseases, including leukemia (38.5%), neuroblastoma (9.6%), and other solid tumors (13.5%). Most transplants were allogeneic (78.8%) from HLA-mismatched relatives (56.1%). Receipt of cyclophosphamide and/or thiotepa during conditioning is associated with the development of TA-TMA [23]. In our cohort, 82.7% of patients received cyclophosphamide and/or thiotepa. Patient outcomes are summarized in Figure 1. Seven patients developed TA-TMA at a median of 76 d from HSCT (range, 20 to 159 d). Six of these patients received allogeneic transplants for leukemia (n = 4) or nonmalignant hematologic disease (n = 2) and one patient received an autologous transplant for neuroblastoma. All TA-TMA patients received cyclophosphamide and/or thiotepa conditioning. Four of the TA-TMA patients required renal replacement therapy, only one of whom survived through the study period. An additional TA-TMA patient died during the study period without having received renal replacement therapy. Twenty patients developed any endotheliopathy at a median of 20 d (range, 4 to 202 d). SOS was the most common endotheliopathy diagnosed, identified in 9 patients at a median of 11 d (range, 4 to 62 d). Five of these patients received allogeneic transplants for leukemia (n = 4) or nonmalignant hematologic disease (n = 1) and the remaining 4 received autologous transplants for neuroblastoma. TA-TMA was additionally diagnosed in just 1 of the patients with SOS and no deaths occurred among the SOS group. In total, 7 patients died during the study period of nonrelapse causes at a median of 144 d (range, 11 to 275 d).

### Echocardiographic Findings

All 52 patients underwent a baseline pre-HSCT echocardiogram. Forty-nine patients had screening echocardiograms performed on d +7, and 33 patients on d +30. Supplementary Table 1 shows the echocardiographic measurements sufficiently obtained and available for analysis at each echocardiogram. Summary descriptive statistics for the echocardiographic measurements are presented in Supplementary Table 2.

Overall, 5/52 (9.6%) patients had pericardial effusions, with 1 patient’s effusion starting prior to HSCT. Pericardial effusions were noted on 3 (6.1%) d +7 echocardiograms and 5 (15.2%) d +30 echocardiograms. All effusions remained small during the 30-d screening period. However, 2 (4%) patients had progression requiring pericardial drainage after the 30-d screening period. Pericardial effusions on either screening echo were associated with an increased risk of later development of TA-TMA (HR: 7.59, 95% CI: 1.80 to 32.00, *P* = .006). Patients with a pericardial effusion on any post-HSCT echocardiogram were 6 times more likely to die compared to patients without a pericardial effusion (95% CI: 1.36 to 27.40, *P* = .018).

**Figure 2.**
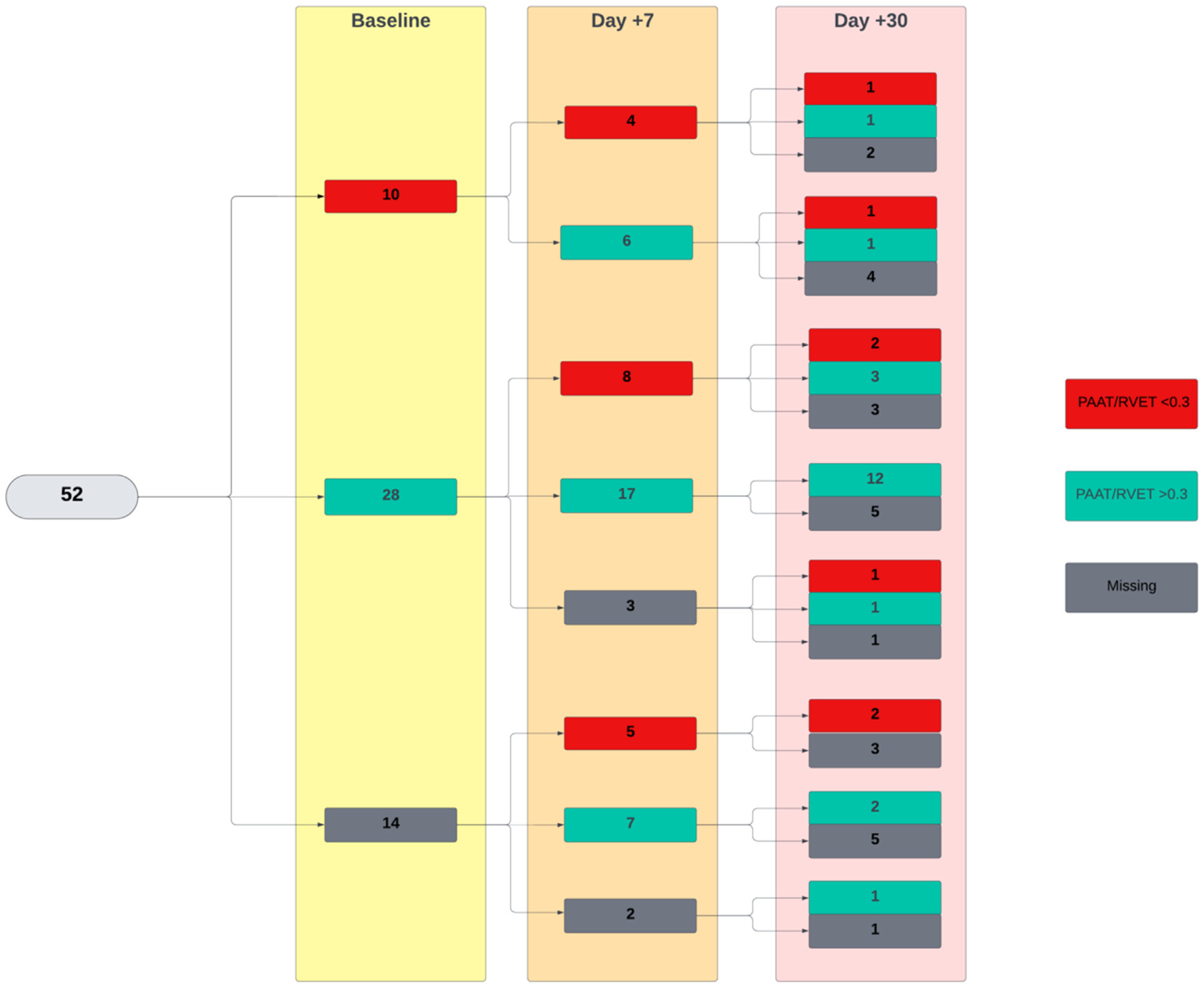
PAAT/RVET ratio, a marker of pulmonary vascular resistance, among the cohort during the screening course. Flow chart depicting the PAAT/RVET ratio of patients throughout the study period. PAAT/RVET < 0.3 is indicative of elevated pulmonary vascular resistance.

No patients were diagnosed with PH during the 30-d period or thereafter. Several patients did have echocardiographic signs of increased pulmonary vascular resistance as evidenced by PAAT/RVET <0.3 (Figure 2). Only one patient had persistent PAAT/RVET <0.3 from baseline throughout the 30-d screening period. Thirteen patients were first noted to have PAAT/RVET <0.3 at d +7 and 2 patients at d +30. Counterintuitively, a 10% decrease in PAAT/RVET was noted in 18 patients at d+7 and was associated with lower risk of endotheliopathy (HR: 0.84, 95% CI: 0.75 to 0.95, *P* = .005). PAAT and PAAT/RVET were not associated with death.

Right ventricular systolic dysfunction (TAPSE z score <−2) was present in 13 (25%) patients total (range [−5.4] to [−2.1]); 8 on baseline echocardiograms, 3 developed RV dysfunction on d +7, and 2 developed RV dysfunction on d +30 (range [−4.3] to (Only −2.1]). one patient had persistent RV dysfunction throughout the screening period with 3 missing the d +30 screening echo. On the other hand, we found that an increase rather than a decrease in ventricular function metrics was associated with our outcomes of interest. A 10% increase from baseline in longitudinal tricuspid annular systolic velocity (tricuspid s’) evaluated at d +30 was identified in 5 patients and associated with an increased risk of TA-TMA (HR: 1.61, 95% CI: 1.08 to 2.38, *P* = .018). A 10% increase from baseline in TAPSE at d +30 was noted in 11 patients and associated with an increased risk of any endotheliopathy (HR: 1.43, 95% CI: 1.08 to 1.90, *P* = .013). Conversely, changes in right ventricular function metrics were not associated with mortality.

Left ventricular systolic dysfunction (LVEF <55%) was present in only one (2%) patient (LVEF 54%) on baseline echocardiogram that recovered (LVEF 61%) at d +7. One (2%) patient developed LV dysfunction on d +7 (LVEF 54%) with no follow-up d +30 echocardiogram. No patients developed new LV dysfunction on d +30. Again, we found that increases rather than decreases in left ventricular function metrics were associated with the outcomes of interest. A 10% increase from baseline in longitudinal mitral annular systolic velocity (mitral s’) evaluated at d +30 was found in 8 patients and associated with an increased risk for TA-TMA and any endotheliopathy diagnosis (HR: 1.70, 95% CI: 1.05 to 2.75, *P* = .031, HR 1.28, 95% CI: 1.05 to 1.57, *P* = .016, respectively). An increase in LV EF of 10% above baseline at d +7 was identified in 10 patients and an increase in lateral mitral annular systolic velocity (s’) at d +30 was noted in 8 patients and both were associated with mortality (HR: 2.15, 95% CI: 1.02 to 4.51, *P* = .043 and HR: 2.87, 95% CI: 1.15 to 7.20, *P* = .025, respectively).

### BNP levels

BNP levels were obtained for 48 patients at baseline and 46 patients at d +7. Seven patients (15%) had an increase in BNP level >50 pg/mL between the 2 measurements, (range 56 to 290 pg/mL). These 7 patients did not have LV or RV dysfunction on their Day +7 echocardiograms and only 2 of them had new PAAT/RVET <0.3. A 50 pg/mL increase from baseline in BNP at d +7 was associated with an increased risk of developing any endotheliopathy (HR: 2.86, 95% CI: 1.15 to 7.08, *P* = .024). Changes in BNP levels were not associated with mortality.

### Predictors of TA-TMA and Any Endotheliopathy

**Figure 3.**
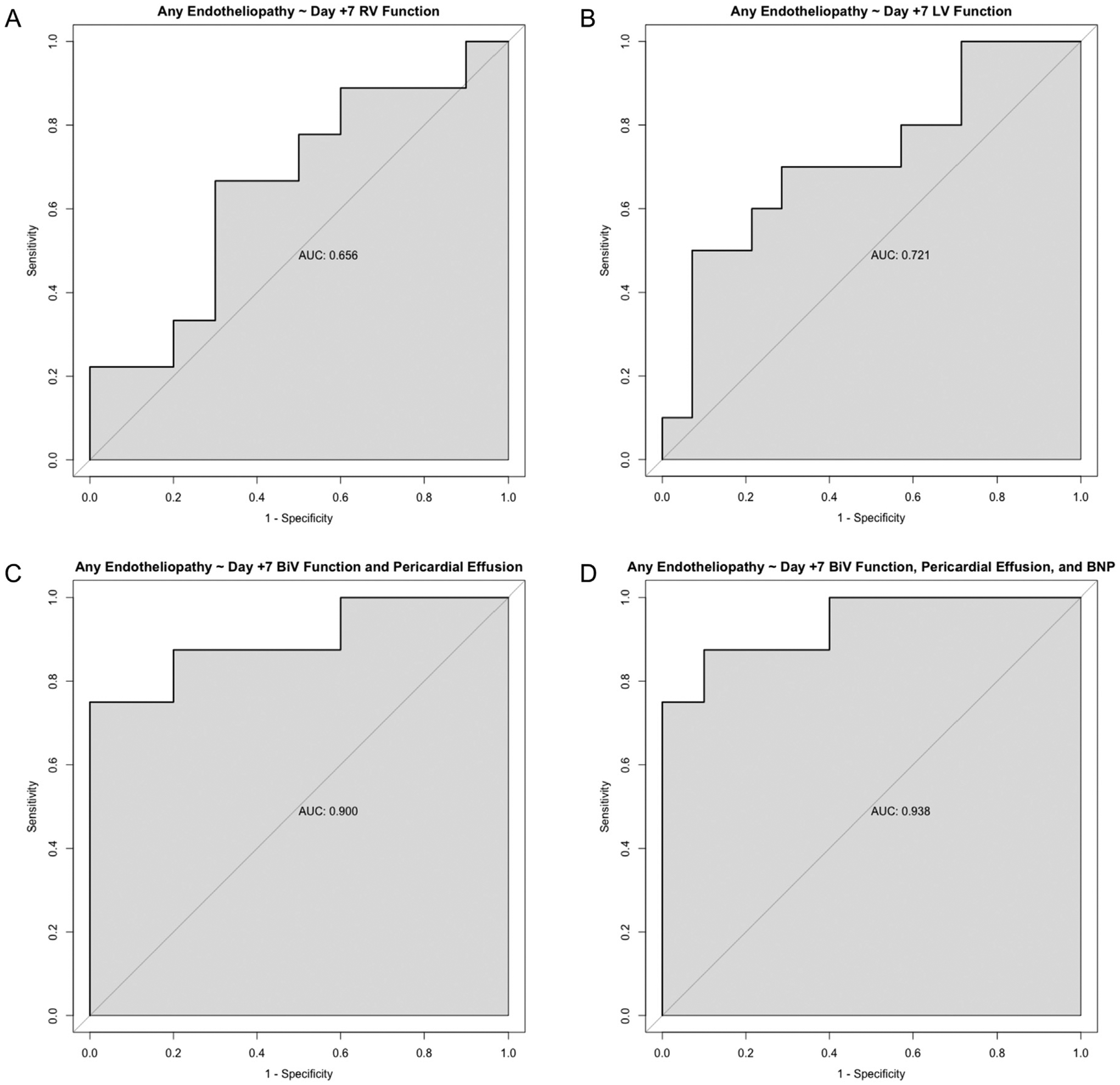
ROC curves of d +7 predictors of endotheliopathy ROC curves depict the predictive performance of echocardiographic metrics of A) RV function, B) LV function, C) biventricular function and pericardial effusion, and D) biventricular function, pericardial effusion, and BNP at d +7 post-HSCT.

We found that combining echocardiographic metrics could more effectively predict endotheliopathy outcomes (Supplemental Table 3). We assessed only metrics at d +7 considering this timing for prediction of endotheliopathy to be of high utility for the clinician. RV function (combining the change in TAPSE and lateral tricuspid S’ changes from baseline) demonstrated relatively poor classification performance, with an AUC of just 0.66 (95% CI: 0.39 to 0.92) (Figure 3). LV performance (combining LV EF and lateral mitral S’ changes from baseline) showed superior performance (AUC: 0.72, 95% CI: 0.50 to 0.94). A model combining biventricular function metrics, and the presence of pericardial effusion had improved performance (AUC: 0.90, 95% CI: 0.74 to 1.00). The best performance was seen with a model incorporating biventricular function metrics, the presence of pericardial effusion, and BNP change from baseline (AUC: 0.94, 95% CI: 0.83 to 1.00).

### Predictors of Mortality

**Figure 4.**
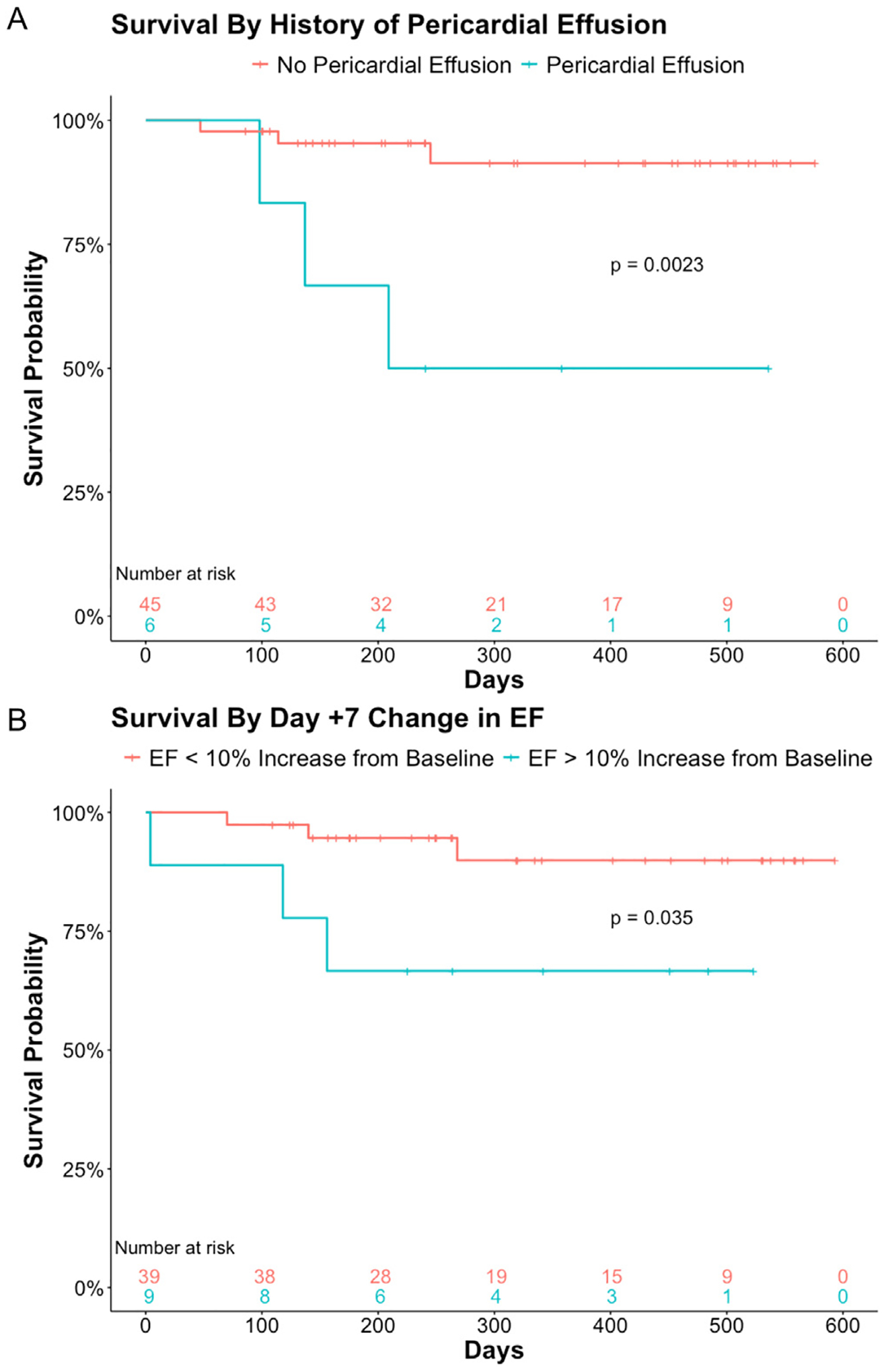
Post-HSCT survival curves Kaplan Meier survival curves of patients post-HSCT who A) develop pericardial effusion in the first 30 d after transplant and, B) who develop a 10% increase in left ventricular ejection fraction at Day +7. Survival time begins at d +30 for pericardial effusions (A) and at d +7 for left ventricular ejection fraction (B).

Pericardial effusions and increases in metrics of systolic left ventricular function, as discussed above, were the only metrics associated with mortality in our cohort (Table 2). Survival curves corroborated these results, demonstrating significant differences in survival after echocardiogram based on changes observed on screening echocardiograms (Figure 4).

## DISCUSSION

In this study we implemented a cardiovascular screening protocol for pediatric HSCT patients using echocardiography and serum BNP levels. Our goal was not only to detect subclinical cardiovascular abnormalities in HSCT patients, but also to understand their association with HSCT complications, specifically TA-TMA and other endotheliopathies. In contrast to common assumptions, increases in metrics of ventricular systolic function, rather than decreases, were associated with TA-TMA, endotheliopathy, and death. In fact, ventricular dysfunction was uncommon in our cohort and we did not encounter any patients with clinically overt pulmonary hypertension. Large pericardial effusions are always concerning in HSCT patients due to the risk of cardiac tamponade and resultant hemodynamic compromise, but our study also highlights the importance of small pericardial effusions in the prediction of endotheliopathy and death.

The association in our study between the increase in ventricular systolic function metrics and poor outcomes is not immediately intuitive. However, endothelial dysfunction and its associated inflammation and neurohormonal alterations can trigger a catecholamine surge that may explain this finding [6,8]. Inflammation also increases capillary permeability and plays a role in the development of pericardial effusions. Apart from inflammation, we must also consider how early endothelial dysregulation may be altering loading conditions on the heart. The endothelium regulates vascular tone and endothelial injury likely alters both the venous return to the heart as well as the arterial afterload on the ventricles. Early adaptations to either increased venous return and/or increased arterial afterload involve alterations to cardiac contractility that augment ventricular function, as described by the Frank-Starling relationship and the Anrep effect [24]. Particularly in the context of TA-TMA and its associated systemic hypertension, our findings may represent early adaptive responses to endothelial injury that precede the later development of cardiovascular dysfunction. While increases in BNP levels were predictive of endotheliopathy in our study they were not related to concomitant clinical signs or symptoms of ventricular dysfunction or PH at this early stage. These findings also suggest that the mechanism of acute cardiovascular adaptation to HSCT-related endotheliopathy is subtle and on the subclinical level, at least in the first 30 d after transplant. This was similarly shown in the study by Rotz et al. where 227 patients were screened with echocardiograms, cardiac troponin-I and soluble suppressor of tumorigenicity 2 (sST2). Pericardial effusion was identified in 27% of patients and LV dysfunction in 6% of patients, yet cardiac troponin-I was elevated in 53% of samples, and sST2 in 38% of samples, suggesting HSCT-related acute cardiac injury may be more prevalent than detected by echocardiography, though sST2 in particular is considered a non-specific marker of endothelial and cardiac injury.^13^ Prior studies have additionally found that early increases in N-terminal pro-BNP (NTproBNP) and troponin-T predict the later development of clinical cardiotoxicity post-transplant [25] More sensitive imaging techniques, such as speckle tracking strain assessment, have also been used to detect post-HSCT cardiac involvement [26,27]. Global longitudinal left ventricular strain was worsened initially in 23 patients after HSCT for sickle cell disease and severe acquired aplastic anemia followed by improvements to baseline by 1 yr [28]. Our findings further support the role for these more sensitive techniques in assessing for subtle changes in ventricular function that may develop early in the disease course of endothelial disorders.

Prior studies have demonstrated notable rates of PH post-HSCT, potentially reflecting an endotheliopathy of the pulmonary vasculature [9,14,29–31]. Dandoy et al. carried out a prospective study utilizing echocardiography on 100 patients on d +7 after HSCT that identified PH in 13% of patients, most of whom were asymptomatic [9]. The difference in the rate of PH between our studies is likely related to the definition of PH utilized. All patients diagnosed with PH in the prior study had “at-risk-for-PH” findings, that is, RV systolic pressure between 35–50% systemic systolic pressure, which is lower than the threshold of greater than 50% that we used and is commonly used for PH diagnosis by echocardiography. The absence of clinical PH in screening studies is not surprising and is probably related to the low incidence of this complication and the timing of the echocardiograms shortly after HSCT. In case reports of PH in HSCT, all patients presented with respiratory symptoms at a median time of 70 d after HSCT. A more sensitive marker of pulmonary vascular disease, the PAAT/RVET, suggested the presence of elevated pulmonary vascular resistance in about a third of patients, yet this finding was surprisingly associated with lower risk of endotheliopathy. The changes in PAAT/RVET were fleeting and mostly unrelated to measures of right ventricular function or PH, suggesting that PAAT/RVET may be reflecting other unknown mechanisms that play a role in endotheliopathy.

Post-HSCT acute cardiac injury is subtle and rarely presents with overt cardiovascular symptoms early on. However, these subtle cardiovascular findings are associated with morbidity and mortality. Therefore, a screening protocol is best suited to detecting these cardiovascular abnormalities. The combination of different biomarkers, e.g. serum biomarkers and imaging-based metrics, is likely necessary for optimal screening. The best predictive model in our study was an additive one incorporating all evidence of cardiovascular involvement on echocardiography. Unfortunately, we did not have the capacity to externally validate our prediction models or formally establish a predictive scoring system. The associations we identified, however, support future work to further validate our findings and to consider the incorporation of echocardiographic findings into a risk score for TA-TMA and other endotheliopathies. Additionally, a number of other risk factors for TA-TMA and endothelial dysfunction are well described among the pediatric HSCT population, including age, indication for transplant, conditioning agents, and infections, among others [19]. Incorporating these factors along with a “cardiovascular risk” derived from echocardiographic and BNP screening would likely add significant predictive power to a risk score. Such a risk score would be of significant utility to aid the clinician in monitoring and managing patients post-HSCT.

Our study is limited by the number of patients who had missing studies and the short duration of follow up. Day +30 echocardiograms were not consistently collected early on in the study period particularly among those patients who were discharged from the hospital prior to day +30, potentially introducing bias to these analyses. We decided to focus on screening studies obtained in the first 30 d because our goal was to identify early markers of endotheliopathy before development of symptoms. Abnormal baseline cardiac biomarkers have been reported in prior studies and may be related to a patient’s indication for transplant or prior medical history [32–34]. Our sample size limited our ability to evaluate the relation between these abnormal baseline findings pre-HSCT and the development of post-HSCT complications. The overall transplant volume at our institution was low during the study period, in part related to the COVID-19 pandemic. We additionally had a relatively unique transplant mix and a large proportion of our patients received cyclophosphamide and/or thiotepa during conditioning, which are independently associated with the development of TA-TMA [23]. Lastly, our prediction modeling utilized forward selection and was not tested on a validation cohort, risking overfitting and highlighting the need for external validation. Despite these limitations, we were able to effectively evaluate the association between a number of novelly investigated echocardiographic metrics and later morbidity and mortality post-HSCT.

In conclusion, our study findings support the premise that early post-HSCT cardiovascular involvement is relatively common on a subclinical level and is related to the development of TA-TMA, endotheliopathy, and death. Therefore, screening programs utilizing a combination of cardiac biomarkers may help to identify at-risk patients. The identification of the best biomarkers suited for this role and the development of a risk score should be the focus of future research.

## Supplementary Material

1

2

Supplementary material associated with this article can be found in the online version at doi:10.1016/j.jtct.2025.11.024.

## Figures and Tables

**Table 1:** Demographics and Pre-transplant Clinical Characteristics

		Total, 52
Age, years (median [IQR])		10.21 [3.91, 15.77]
**Age group**	1–4 yr	18 (34.6)
	5–12 yr	16 (30.8)
	>13 yr	18 (34.6)
**Sex, female**		18 (34.6)
**Race**	White	39 (75.0)
	Black or African American	2 (3.8)
	Asian	8 (15.4)
	Native Hawaiian or Pacific Islander	1 (1.9)
	More than one race	2 (3.8)
**Ethnicity, Hispanic or Latino**	27 (51.9)	
**BMI (median [IQR])**	18.18 [16.28, 22.95]	
**BMI Classification**	Underweight	4 (9.5)
	Normal	24 (57.1)
	Overweight	8 (19.0)
	Obese	6 (14.3)
**Indication for transplant**	Malignant disease	32 (61.5)
	*Leukemia*	*20 (38.5)*
	*Neuroblastoma*	*5 (9.6)*
	*Other solid tumor*	*7 (13.5)*
	Non-malignant hematologic disease	11 (21.2)
	Inborn Errors of Immunity	8 (15.4)
	Inborn Errors of Metabolism	1 (1.9)
**Transplant source**	Allogeneic	41 (78.8)
	Autologous	11 (21.2)
**HLA matching**	HLA-identical sibling	9 (22.0)
	HLA-mismatched relative	23 (56.1)
	HLA-matched unrelated	4 (9.8)
	HLA-mismatched unrelated	5 (12.2)
**Cyclophosphamide/Thiotepa conditioning**	Cyclophosphamide only	7 (13.5)
	Thiotepa only	28 (53.8)
	Cyclophosphamide + thiotepa	8 (15.4)
	Neither cyclophosphamide nor thiotepa	9 (17.3)
**Recipient CMV status, positive**		32 (61.5)
**Lansky/Karnofsky score**	100	24 (46.2)
	90	18 (34.6)
	≤80	10 (19.2)

Demographics and pre-transplant clinical characteristics of all patients in the cohort.

**Table 2:** Predictors of Risk of TA-TMA, Endotheliopathy, and Death

		TA-TMA		Any Endotheliopathy		Death	
		Hazard Ratio	*P* value	Hazard Ratio	*P* value	Hazard Ratio	*P* value
**Pericardial Effusion** (Day +7 and/or Day +30)	7.59 (1.80–32.00)	0.006	1.68 (.74–3.83)	0.21	6.11 (1.36–27.40)	0.018	
**Pulmonary Hypertension**							
EI	Day +7	1.14 (0.74–1.76)	.55	1.10 (0.66–1.84)	.72	1.07 (1.00–1.13)	.038
	Day +30	1.29 (0.62–2.68)	.50	0.81 (0.54–1.21)	.30	1.03 (0.90–1.04)	.36
PAAT/RVET	Day +7	0.93 (0.73–1.20)	.59	0.84 (0.75–0.95)	.005	0.95 (0.75–1.20)	.67
	Day +30	1.01 (0.82–1.25)	.93	1.02 (0.85–1.23)	.84	1.05 (0.83–1.33)	.67
**RV Function**							
TAPSE	Day +7	0.83 (0.59–1.16)	.27	1.25 (0.98–1.61)	.08	0.93 (0.61–1.42)	.74
	Day +30	0.91 (0.70–1.18)	.46	1.43 (1.08–1.90)	.013	1.00 (0.67–1.49)	.99
Lateral tricuspid s’	Day +7	0.80 (0.58–1.11)	.19	1.15 (0.89–1.48)	.27	0.88 (0.61–1.28)	.51
	Day +30	1.61 (1.08–2.38)	.018	1.10 (0.86–1.42)	.44	1.48 (0.89–2.46)	.13
Lateral tricuspid e’	Day +7	0.96 (0.73–1.26)	.78	1.10 (0.85–1.43)	.47	0.99 (0.93–1.06)	.82
	Day +30	1.40 (0.93–2.10)	.11	1.33 (0.95–1.85)	.10	1.04 (0.97–1.12)	.26
**LV Function**							
LVEF	Day +7	1.30 (0.61–2.78)	.50	0.93 (0.60–1.44)	.74	2.15 (1.02–4.51)	.043
	Day +30	0.70 (0.15–3.34)	.66	0.71 (0.40–1.26)	.24	0.83 (0.26–2.69)	.76
Lateral mitral s’	Day +7	1.11 (0.98–1.26)	.09	1.06 (0.95–1.18)	.29	1.13 (0.98–1.31)	.09
	Day +30	1.70 (1.05–2.75)	.031	1.28 (1.05–1.57)	.016	2.87 (1.15–7.20)	.025
Lateral mitral e’	Day +7	1.21 (0.63–2.32)	.57	1.22 (0.90–1.66)	.20	1.05 (0.98–1.12)	.16
	Day +30	0.95 (0.70–1.30)	.75	1.20 (1.02–1.42)	.025	1.01 (0.98–1.04)	.59
4C longitudinal Strain	Day +7	0.72 (0.34–1.51)	.38	1.03 (0.65–1.64)	.90	1.04 (0.98–1.09)	.19
	Day +30	0.79 (0.49–1.28)	.33	0.80 (0.56–1.13)	.21	1.03 (0.94–1.13)	.57
**BNP**	Day +7	3.06 (0.53–17.80)	.21	2.86 (1.15–7.08)	.024	3.51 (0.78–15.74)	.10

4C = 4 chamber, BNP = brain natriuretic peptide, EI = eccentricity index, LV EF = left ventricular ejection fraction, PAAT/RVET = pulmonary artery acceleration time/right ventricular ejection time, TAPSE = tricuspid annular plane systolic excursion, TA-TMA = transplant-associated thrombotic microangiopathy Risk of TA-TMA and any endotheliopathy was assessed with competing risk regression analyses accounting for death as a competing event. Risk of death was assessed with Cox proportional hazards regression. Hazard ratios describe the risk of the respective outcomes associated with a 10% increase in each predictor from baseline, the presence of pericardial effusion on either d +7 or d +30, and a 50 pg/dL increase in BNP from baseline.
