# Supplementary material for: Early Echocardiographic and Serum Biomarkers Predict Thrombotic Microangiopathy, Endotheliopathy, and Survival After Pediatric Hematopoietic Stem Cell Transplant
Source: Transplant Cell Ther. Author manuscript; Available in PMC 2026 Jun 22. (PMC13285009; doi:10.1016/j.jtct.2025.11.024)
Supplement: 1 [file NIHMS2186790-supplement-1.docx]

Supplemental Figure 1: Echocardiographic metrics

Schematic of the echocardiographic metrics evaluated as predictors of transplant-associated thrombotic microangiopathy, endotheliopathies, and death.

Supplemental Table 1. Summary of available predictor measurements in cohort at each follow up

|  | **Baseline (n=52)** | **Day +7 (n=49)** | **Day +30 (n=33)** |
| --- | --- | --- | --- |
| **EI** | 43 | 39 | 26 |
| **PAAT / RVET** | 38 | 35 | 23 |
| **TAPSE** | 41 | 44 | 30 |
| **Lateral tricuspid s’** | 25 | 20 | 14 |
| **Tricuspid E/A^*^** | 39 | 12 | 6 |
| **Lateral tricuspid e’** | 25 | 15 | 14 |
| **Tricuspid E/lateral tricuspid e’^*^** | 23 | 10 | 6 |
| **LV EF** | 52 | 48 | 32 |
| **Lateral mitral s’** | 30 | 25 | 15 |
| **Mitral E/A^*^** | 39 | 10 | 5 |
| **Lateral mitral e’** | 30 | 19 | 13 |
| **Mitral E / lateral mitral e’^*^** | 30 | 6 | 3 |
| **4C longitudinal strain** | 33 | 25 | 17 |
| **BNP** | 48 | 46 | 15 |

**^*^**Not assessed as a predictor due to insufficient data. Mitral and tricuspid valve inflow and tissue Doppler measurements were lacking secondary to the fusion of the early and late velocity waves in most studies.

4C = four chamber, BNP = brain natriuretic peptide, EI = eccentricity index, LV EF = left ventricular ejection fraction, PAAT/RVET = pulmonary artery acceleration time/right ventricular ejection time, TAPSE = tricuspid annular plane systolic excursion

Supplemental Table 2. Summary statistics of predictors of outcome in pediatric HSCT patients

|  | Baseline  (n=52) | Day +7  (n=49) | Day +30  (n=33) |
| --- | --- | --- | --- |
| **Pericardial Effusion (n (%))** | 1 (1.9) | 3 (6.1) | 5 (15.2) |
| **Pulmonary hypertension** | | | |
| >50% systemic RV pressure (n (%)) | 0 (0) | 0 (0) | 0 (0) |
| EI | 0.98 [0.91-1.08] | 1.01 [0.91-1.06] | 0.95 [0.87-1.05] |
| PAAT/RVET | 0.36 [0.30-0.47] | 0.32 [0.27-0.37] | 0.37 [0.31-0.43] |
| **Right ventricular function** | | | |
| TAPSE (mm) | 20.2 [17.5-23.6] | 22.0 [18.8-24.8] | 22.2 [19.4-24.3] |
| TAPSE z-score | -0.5 [-1.8-1.7] | 1.1 [-0.8-2.7] | 1.3 [-0.4-3.5] |
| 10% increase in TAPSE (n (%)) | N/A | 14 (28.6) | 11 (33.3) |
| Lateral tricuspid s’ (cm/s) | 13 [11-13] | 13 [12-15] | 13 [11-15] |
| Tricuspid E/A | 1.28 [1.05-1.65] | 1.17 [1.11-1.30] | 1.25 [1.02-1.44] |
| Lateral tricuspid e’ (cm/s) | 13 [11-16] | 14 [13-16] | 12 [11-14] |
| Tricuspid E/lateral tricuspid e’ | 0.05 [0.03-0.06] | 0.04 [0.04-0.05] | 0.04 [0.04-0.06] |
| **Left ventricular function** | | | |
| LV EF (%) | 64.8 [62-68] | 64.5 [62.2-67.2] | 63.8 [61.3-69.2] |
| LV EF ≤ 55% (n (%)) | 1 (1.9) | 3 (6.1) | 0 (0) |
| 10% increase in LV EF (n (%)) | N/A | 9 (18.4) | 3 (9.1) |
| Lateral mitral s’ (cm/s) | 9 [7-10] | 9 [8-11] | 9 [7-10.5] |
| Mitral E/A | 1.64 [1.40-1.97] | 1.45 [1.17-1.63] | 1.66 [1.11-1.70] |
| Lateral mitral e’ (cm/sec) | 15.5 [13.0-18.0] | 14 [12-16] | 13 [11-17] |
| 4C Longitudinal Strain (%) | 20.7 [18.6-23.1] | 20.1 [17.2-23.5] | 21.3 [20-23.6] |
| BNP (pg/dL) | 9.0 [0.0, 13.5] | 11.5 [0.0, 46.0] | 12.0 [3.0, 20.0] |

Continuous metrics are depicted as median [IQR] and categorical as n (%). 4C = four chamber, BNP = brain natriuretic peptide, EI = eccentricity index, LV EF = left ventricular ejection fraction, PAAT/RVET = pulmonary artery acceleration time/right ventricular ejection time, TAPSE = tricuspid annular plane systolic excursion

Supplemental Table 3. Predictive performance of day +7 metrics for any endotheliopathy outcome

|  | **AUC** | **95% Confidence Interval** | **IBS** | **95% Confidence Interval** |
| --- | --- | --- | --- | --- |
| **TAPSE** | 0.67 | 0.48-0.86 | 0.20 | 0.16-0.26 |
| **Lateral tricuspid s'** | 0.61 | 0.34-0.87 | 0.20 | 0.15-0.26 |
| **RV Function (TAPSE + Lateral tricuspid s')** | 0.66 | 0.39-0.92 | 0.19 | 0.13-0.26 |
| **LV ejection fraction** | 0.51 | 0.33-0.68 | 0.24 | 0.20-0.27 |
| **Lateral mitral s’** | 0.77 | 0.55-0.98 | 0.22 | 0.19-0.26 |
| **LV Function (LV ejection fraction + lateral mitral s’)** | 0.72 | 0.50-0.94 | 0.18 | 0.11-0.24 |
| **Pericardial effusion** | 0.48 | 0.40-0.57 | 0.24 | 0.21-0.27 |
| **BNP** | 0.65 | 0.51-0.78 | 0.21 | 0.15-0.27 |
| **Biventricular function (TAPSE + lateral tricuspid s’ + LV ejection fraction + lateral mitral s’)** | 0.91 | 0.77-1.00 | 0.10 | 0.02-0.18 |
| **Biventricular function + pericardial effusion** | 0.90 | 0.74-1.00 | 0.10 | 0.05-0.19 |
| **Biventricular function + pericardial effusion + BNP** | 0.94 | 0.83-1.00 | 0.10 | 0.00-0.20 |

Echocardiographic metrics and BNP levels were included in the predictive models as continuous variables referencing percent change from baseline echo. Pericardial effusions were included as a binary variable (present/absent). AUC = area under receiver operating characteristic. IBS = integrated Brier score.
