# Supplementary material for: Early Echocardiographic and Serum Biomarkers Predict Thrombotic Microangiopathy, Endotheliopathy, and Survival After Pediatric Hematopoietic Stem Cell Transplant
Source: Transplant Cell Ther. Author manuscript; Available in PMC 2026 Jun 22. (PMC13285009; doi:10.1016/j.jtct.2025.11.024)
Supplement: 2 [file NIHMS2186790-supplement-2.pdf]

RV Function

- TAPSE: distance traveled by tricuspid annular plane in systole
  ↓ = ↓ **RV systolic function**
- Lateral tricuspid s’: peak systolic velocity of tricuspid annulus
  ↓ = ↓ **RV systolic function**
- Lateral tricuspid e’: early diastolic velocity of tricuspid annulus
  ↓ = ↓ **RV diastolic function**

LV Function

- EF: fraction of volume ejected from LV in systole
  ↓ = ↓ **LV systolic function**
- Lateral mitral s’: peak systolic velocity of mitral annulus
  ↓ = ↓ **LV systolic function**
- Lateral mitral e’: early diastolic velocity of mitral annulus
  ↓ = ↓ **LV diastolic function**
- 4 chamber longitudinal strain: metric of myocardial contractility
  ↓ (more negative) = ↑ **LV systolic function**

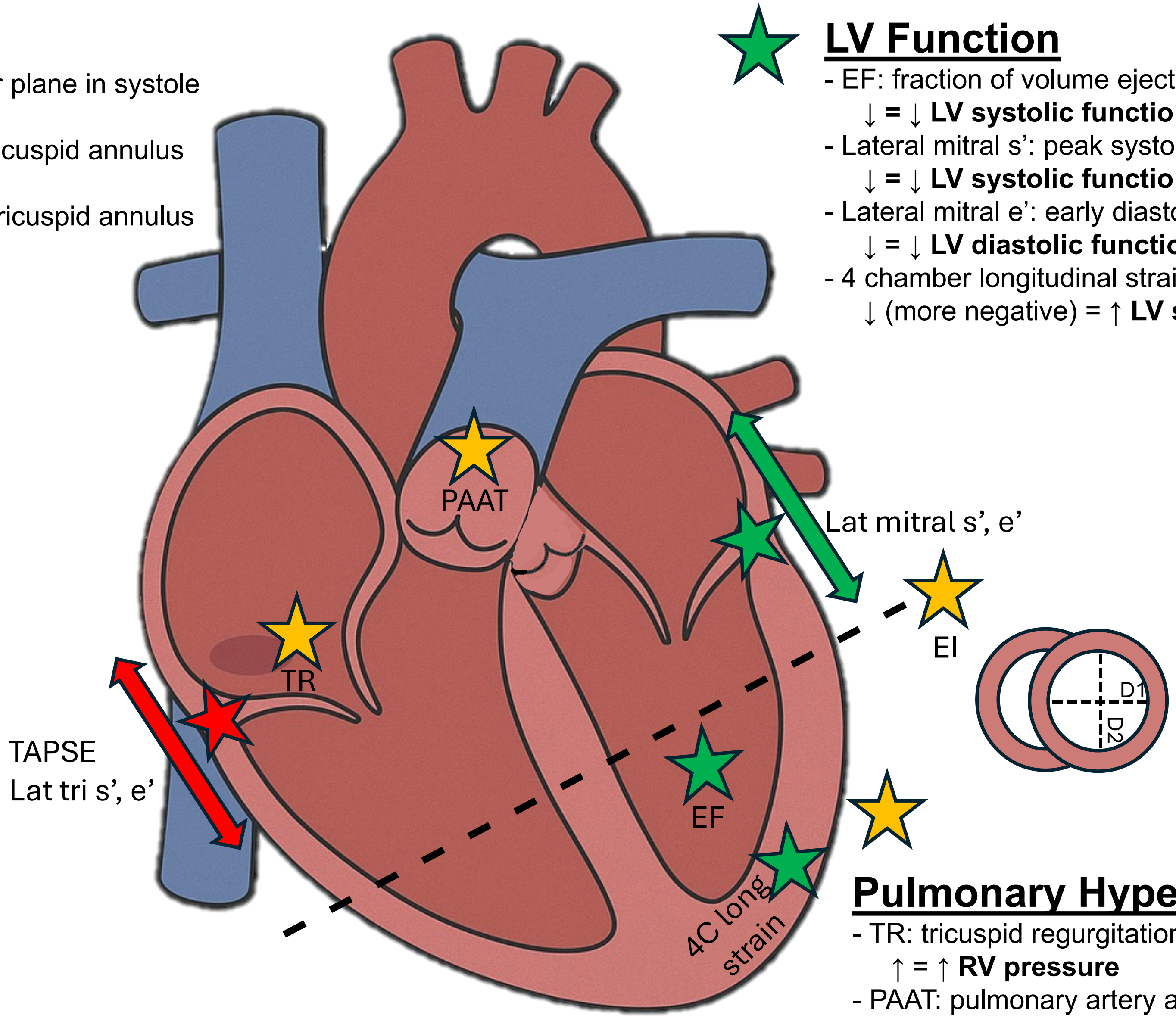

Pulmonary Hypertension

- TR: tricuspid regurgitation jet peak velocity
  ↑ = ↑ **RV pressure**
- PAAT: pulmonary artery acceleration time
  ↓ = ↑ **Pulmonary Vascular Resistance**
- Eccentricity index: ratio of LV dimensions D2:D1, reflective of septal flattening due to RV pressure
  ↑ = ↑ **RV pressure**
